# Supplementary material for: Current and future distribution of Ixodes scapularis ticks in Québec: Field validation of a predictive model
Source: PLoS One. 2022 Feb 3;17(2):e0263243. doi: 10.1371/journal.pone.0263243 (PMC8812838; doi:10.1371/journal.pone.0263243)
Supplement: S1 File — (DOC) [file pone.0263243.s001.doc]

Access to Québec data surveillance needs a formal request to government of Québec. Access to model prediction needs a request to the authors of the publication of Leighton *et al.* (2012)
