# Supplementary material for: Current and future distribution of Ixodes scapularis ticks in Québec: Field validation of a predictive model
Source: PLoS One. 2022 Feb 3;17(2):e0263243. doi: 10.1371/journal.pone.0263243 (PMC8812838; doi:10.1371/journal.pone.0263243)
Supplement: Attachment — Submitted filename: QuebecTickEstablishment_submission_20210428_v02.doc [file pone.0263243.s002.doc]

**TITRE**

Current and future distribution of *Ixodes scapularis* ticks in Québec: field validation of a predictive model previously developed in Canada

**AUTHORS**

Marion Ripoche^1*^, Catherine Bouchard^2^, Alejandra Irace-Cima^1^, Patrick Leighton^3^, Karine Thivierge^4^

^1^ Direction des risques biologiques et de la santé au travail, Institut National de Santé Publique du Québec, 190 boulevard Crémazie Est, Montréal, Québec, Canada, H2P 1E2

^2^ Public Health Risk Sciences Division, National Microbiology, Public Health Agency of Canada, 3200 rue Sicotte, Saint-Hyacinthe, Québec, Canada, J2S 2M2

^3^ Department of Pathology and Microbiology, Faculty of Veterinary Medicine, University of Montréal, 3200 rue Sicotte, Saint-Hyacinthe, Québec, Canada, J2S 2M2

^4^ Laboratoire de santé publique du Québec, Institut national de santé publique du Québec, 20045 Chemin Sainte-Marie, Sainte-Anne-de-Bellevue, Québec, Canada, H9X 3R5

*Corresponding author: Marion Ripoche

Address : Direction des risques biologiques et de la santé au travail, Institut National de Santé Publique du Québec, 190 boulevard Crémazie Est, Montréal, Québec, Canada, H2P 1E2

Email: marion.ripoche@inspq.qc.ca

**ABSTRACT (250 mots max)**

**Background.** Lyme disease is increasing in Québec and is related to *Ixodes scapularis* tick distribution. A time-to-establishment model developed in 2012, by Leighton and colleagues, predicted the year of tick population establishment for each municipality in eastern Canada, until 2100. **Objective.** To validate if this model correctly predicted tick distribution in Québec. **Methodology.** For each municipality, the predicted tick establishment was compared to field data from active tick surveillance (2010-2018) using two criteria: i) the detection of at least one tick and ii) the presence of the three stages; using kappa coefficient and Chi-square test. The speed of tick establishment and the increase of exposed human population by 2100 were predicted with the time-to-establishment model. **Results.** Field observations were consistent with model predictions. Ticks were detected on average 3 years after the predicted year. The probability of tick detection is significantly higher after the predicted year than before (61% vs 27% of collections). The trend is similar for the three stages detection (16% vs 9% of collections). The average tick progression was estimated with the model at 18 km/year in Québec, but not homogenous everywhere, with 90% of the human population exposed by 2032. **Conclusion.** The validation of the time-to-establishment model using the field data confirmed that it could be used to project *I. scapularis* progression in Quebec, and consequently the Lyme disease risk for the next decades. This will help public health authorities anticipate and adapt preventive measures, especially in areas not affected yet by Lyme disease.

**Key words:** *Borrelia burgdorferi*, projection, emergence, climate change, tick-borne disease, spread

**INTRODUCTION**

Lyme disease, caused by *Borrelia burgdorferi* sensu lato complex (hereafter shortened to *B. burgdorferi*), is the most common vector-borne disease in North America (1). The number of locally acquired human cases increased from 144 in 2009 to 2636 cases in 2019, in Canada (2). In Eastern Canada, the incidence of Lyme disease is increasing because of the northern expansion from the United States, of the blacklegged tick (*Ixodes scapularis*), the main vector of *B. burgdorferi*, as a consequence of climate change and, the presence of suitable habitat (deciduous or mixed forests) and of the host communities (white-footed mice - *Peromyscus leucopus* and white-tailed deer - *Odocoileus virginianus*) (3,4). In Québec, a province in eastern Canada, the integrated Lyme disease surveillance program, with a human and acarological component, provides standardized baseline data to follow the spread of Lyme disease (5), which has been a notifiable disease in Québec since 2003. Passive tick surveillance programs have been conducted in Quebec since 1990 and consist of submissions of ticks found on people or pets, by medical and veterinary health professionals. Active surveillance programs have been conducted in Quebec since 2007 and consists of collecting ticks from different sites in the environment by the dragging method (5,6). In Quebec, the number of locally acquired human cases increased, from 2 cases in 2008 to 381 by 2019 (7). Past active surveillance informs us that blacklegged ticks that were initially detected south of the St. Lawrence River are now frequently detected to the north of the river (8–10).

The distribution of established *I. scapularis* tick populations is a well-known proxy of human Lyme disease risk, as *B. burgdorferi* is then likely to circulate between tick and host populations and become endemic (11–13). The lifecycle of ticks takes place over two years; briefly larvae in summer, nymphs next spring, and adults in autumn) (11). Finding the three stages at one collection site during the same year suggests that they are not of the same generation and that at least one reproduction cycle has occurred (12). Another study suggested that only one tick collected by the flannel method for three person-hours would be sufficient to define the establishment of a tick population (13). In Québec, public health authorities use the criterion of the three stages in one year to determine if a tick population has established (5). Currently, ticks can be found throughout Québec, due to “adventitious” ticks brought by migrating birds or terrestrial animals but established tick populations have only been identified in the southern parts of Quebec (14,15). Detecting and anticipating the location of newly established tick populations would help public health authorities develop local prevention measures.

Active surveillance provides useful information about the distribution of ticks, but will not detect and predict all established tick populations in space and time. A predictive risk map of future tick occurrence according to various climate change scenarios was produced based on a temperature threshold suitable for tick establishment (cumulative annual number of degree days >0⁰C) (16,17). This model was also used by Simon et al. for a current and future risk index for *B. burgdorferi* in Quebec, using potential distributions of ticks and white-footed mice based on climatic and landscape variables (18). These models give interesting information about potential tick distribution in the environment but are too complex to be easily used by public health authorities. Another model predicted the year of tick establishment per municipality in eastern Canada, using a parametric survival regression model based on passive surveillance data and environmental factors, such as temperature, elevation, annual rainfall, and local and long-distance dispersal of ticks (19). This model gave a predictive map of tick establishment per year with an estimated speed of spread of 46 km/year. This “time-to-establishment” model could be a simple and useful tool for public health authorities to estimate the change in risk per municipality and year. Field data from Ontario agreed with this model’s prediction (20), but no validation with field data from Québec has yet been investigated.

The objective of our study was to assess the concordance between field observations from active tick surveillance (2010-2018) and the predicted tick establishment by the model of Leighton et al. (19). We investigated whether the model was able to predict the detection of the three stages or at least one tick by active surveillance. We then used the model projection to 2100 to investigate the pattern and the speed of tick spread and the increase of human exposure to ticks in the next decades in Quebec.

**MATERIALS AND METHODS**

**Data**

- **Study area.** Québec is in the eastern part of Canada, in between the provinces of Ontario and New Brunswick and bordering the United States. It is the largest and the second-most populated province in Canada, with 8,164,361 inhabitants distributed over 1,356,625 km^2^ (21). There are 1,226 municipalities, or census subdivisions (CSDs), with a median area of 100 km^2^ and an average population of 6,639 inhabitants (ranging from 5 to 1,650,000; median = 1,142). For each municipality included in the study, we estimated the population size and the area (km^2^) from the 2016 census data (21).
- **Model predictions.** The predicted year of tick establishment for *I. scapularis* per municipality came from a parametric survival regression model developed for eastern Canada with passive surveillance data (1990-2008) and environmental factors, such as temperature (degree days > 0°C), elevation, annual rainfall, and local and long-distance dispersal of ticks (19). For each municipality, there was a mean predicted year, with a lower predicted year (reflecting the fastest progression of ticks) and an upper predicted year (reflecting the slowest progression of ticks), based on the maximum and minimum temperatures observed in each CSD between 1991 and 2008 to account for potential climate change (19).
- **Active tick surveillance data**. Tick distribution comes from annual Lyme disease surveillance activities in Québec, carried out jointly by the Institut national de santé publique du Québec (INSPQ), the Ministère de la Santé et des Services Sociaux (MSSS), the Public Health Agency of Canada (PHAC), and the University of Montréal (5,6). A standardized procedure was used from 2010 to 2018, with drag sampling carried out between June and September in different woodlands that are open to the public. Host-seeking (or questing) ticks were collected by dragging a 1 m^2^ flag of white flannel over the forest floor, in the morning or early afternoon (22), covering 2000 m^2^ per site. Drag sampling did not occur on rainy days or when the forest floor was wet. Every 25 m, the flag was inspected, and collected ticks were sent to the Laboratoire de Santé Publique du Québec (LSPQ) for tick species and life stage identification, using taxonomic keys for larvae, nymphs and adults (23,24). Surveillance data of 2019 and 2020 were not available when we did the analyses.

**Model validation**

- **Established tick population.** The model developed by (19) (25,26) was designed to predict the year of tick population establishment, based on the detection of at least two adults or one immature tick, by active surveillance for two consecutive years. These definitions of tick population establishment are not currently used by Quebec’s public health authorities to assess the risk. To understand how to use this model for public health in Quebec, we decided to compare predictions with two indicators of active surveillance: 1) detection of the three tick stages in one year, which is the Quebec surveillance definition of an established tick population (5); 2) detection of at least one tick, which is a broader definition of potential tick population establishment (25). Concerning the model prediction, we used the lower, mean and upper predicted year of the model to test the sensitivity of the model. We considered that, according to the model, tick populations were supposed to be established in a municipality from the predicted year by the model.
- **Statistical analysis.** We investigated two measures of concordance between observations and predictions, using the kappa coefficient of determination and the matched McNemar Chi-square test, with a significance level of p<0.05. The kappa statistic was interpreted as follows: > 0.75 as excellent, 0.40 to 0.75 as fair to good, and < 0.40 as poor agreement. Analyses were carried out using the *kappa* and *mcnemar.test* function in R version 4.0.2 (R Development Core Team, Vienna, Austria) (27).
  - **Concordance per collection:** Do we find tick populations when and where the model predicted established tick populations? For each tick collection, in a given municipality and year, (n=444 tick collections), we determined: i) whether all three tick stages were detected (yes/no) in field data; ii) whether some ticks were collected (yes/no) in field data; and iii) whether the model predicted established tick populations (yes/no) in this municipality the year of collection. After the predicted year, tick population are supposed to be established in the municipality, according to the model, so ticks are expected in the field data. We compared the result of active surveillance with model predictions.
  - **Concordance over the study period:** Have we already found tick populations between 2010 and 2018 where the model predicted established tick populations before 2018? Because the dragging method has a low sensitivity (25,26), we aggregated surveillance data per municipality over the study period, cumulating active surveillance data from 2010 to 2018. For each municipality with active surveillance during 2010 – 2018 (n=231 CSDs), we determined: i) whether all three tick stages were detected at least once before 2018 (yes/no) in field data; ii) whether some ticks were collected before 2018 (yes/no) in field data; and iii) whether the model predicted established tick populations in 2018 (yes/no). The model predicted an established tick population in a municipality if the predicted year was 2018 or before. We compared the result of active surveillance with model predictions.

We also estimated the delay between the exact predicted year and the first detection of the three stages or the detection of at least one tick in a municipality by active surveillance.

**Analysis of projection until 2100 in Quebec.**

- **The annual speed of tick progression** for Quebec was calculated using the model of Leighton et al. (19) per year, as the difference of the square roots of predicted established tick area between two consecutives years [speed (km/year) = √ (area year n) - √ (area year n-1)]. For that, we calculated for each year the sum of areas of municipalities where the model predicted tick population establishment. We cumulated the annual predicted areas, from 2006 to 2100.
- **The human population exposed to tick** was calculated, for each year, as the number of inhabitants living in the municipalities where the model predicted established tick population. We cumulated the annual predicted exposed human population, from 2006 to 2100, and calculated the annual percentage of exposed people across Quebec population, based on the 2016 census data (21).
- **The predictive map** of established tick populations was displayed with prediction data from Leighton et al. (19) to highlight regional specificities that can be expected in the coming years. We used QGIS software version 3.4.9 (QGIS Geographic Information System).

**RESULTS**

**Data**

- **Model predictions.** From Québec’s 1,226 CSDs, the model of Leighton et al. (19) was able to predict the year of tick population establishment in 1,180 CSDs since 46 were excluded from the study because of a lack of environmental data. The predicted spread of tick establishment was centrifugal from the southern border of Québec with Ontario and the United States (Figure 1). For 44% of the municipalities, the tick population establishment was predicted before 2018 (540 municipalities according to the mean predicted year; 284 and 651 municipalities according to the upper and lower predicted year).

Figure 1 Tick collections in Quebec municipalities in 2010-2018 and expected tick population establishment by 2018. Active surveillance for blacklegged ticks in Quebec from 2010 to 2018 found three types of municipalities: (1) detection of three tick stages (black stars), (2) presence of at least one tick (black triangles), and (3) no tick detection (white circles). The grey area represents municipalities with the expected tick population establishment by 2018 according to the lower, mean, and upper predicted year (colored in light, medium, and dark grey, respectively).

- **Active tick surveillance.** Between 2010 and 2018, ticks were sampled in 231 of the CSDs, for a total of 444 tick collections - municipalities were sampled twice on average during the study period (range: 1 to 7 samples per municipality). At least one *I. scapularis* tick was detected in 218 collections (49%), including 52 (12%) with detection of all three stages. In the remaining 226 collections (51%), no ticks were detected by the dragging method. Overall, in 2018, at least one *I. scapularis* was detected in 122 municipalities (53%), including 35 (15%) with detection of the three stages in one year. No ticks were detected in the remaining 109 collected municipalities (47%). Most of the municipalities with ticks were located in the mean predicted year area of the model (115/122 with at least one tick, 30/35 with the three stages). The other 5 municipalities with the three stages were located near the model boundary.

**Concordance per collect.**

- **Detection of three tick life stages.** The probability of detecting the three tick life stages during one collection by active surveillance is significantly higher when the model predicts an established tick population (i.e., the collection is made in a municipality after the predicted year of tick establishment for this municipality). The three stages were detected in 16% (47/289) of the collections that took place after the mean predicted year, but in 3% (5/155) of the collections that took place before the mean predicted year (lower year: 3% vs 1%; upper year: 16% vs 9%; p<0.001) (Table 1). The agreement between the three stages observation and prediction is low but significant, and better with the mean or upper predicted year (kappa = 0.09 [IC95% 0.05-0.13], p<0.001). The first detection of the three stages by active surveillance was on average three years after the mean predicted year (mean = 3.32; sd = 2.91; range = [-5; 8]) (Table 2). The three stages were detected only once before the lower predicted year, but with only one year earlier.
- **Detection of at least one tick.** We observed the same trends as with the three stages. Ticks were detected in 61% (176/289) of the collections that took place after the mean predicted year, and 27% (42/155) of tick collections that took place before the mean predicted year (lower year: 52% vs 28%; upper year: 63% vs 41%; p<0.001) (Table 1). The best fit was with the mean predicted year (kappa = 0.30 [95%CI: 0.22-0.38], p<0.001). The first detection of ticks by active surveillance was on average two years after the mean predicted year (presence of ticks: 2.4; sd = 3.73; range = [-12;9]) (Table 2).

**Table 1. Concordance between field data and model predictions per year. Number and percentage of tick collections based on surveillance results (no detection, tick presence, three stages) and time of collection relatively to prediction (before or after the lower, mean and upper predicted year).**

|  |  | |  | **Number (%) of tick collections (n=444)** | | | | **Kappa [IC 95%]**  **A: Tick presence**  **B: Three stages** | **McNemar test**  **p-value** |
| --- | --- | --- | --- | --- | --- | --- | --- | --- | --- |
|  | | **Established tick pop.**  (Year of tick collection) | **Total** | | **No detection** | **Tick presence** | **Three stages^1^** |  |  |
| **Lower predicted year** | **Expected** (After predicted year) | | 378 | | 179 (48%) | 199 (52%) | *51 (3%)* | A: 0.11 [0.05-0.18] | <0.001 |
|  | **Not expected** (Before predicted year) | | 66 | | 47 (72%) | 19 (28%) | *1 (1%)* | B: 0.03 [0.02-0.05] | <0.001 |
| **Mean predicted year** | **Expected** (After predicted year) | | 289 | | 113 (39%) | 176 (61%) | *47 (16%)* | A: 0.30 [0.22-0.38] | <0.001 |
|  | **Not expected** (Before predicted year) | | 155 | | 113 (78%) | 42 (27%) | *5 (3%)* | B: 0.09 [0.05-0.13] | <0.001 |
| **Upper predicted year** | **Expected** (After predicted year) | | 160 | | 58 (37%) | 102 (63%) | *26 (16%)* | A: 0.21 [0.12-0.30] | <0.001 |
|  | **Not expected** (Before predicted year) | | 284 | | 168 (59%) | 116 (41%) | *25 (9%)* | B: 0.09 [0.01-0.17] | <0.001 |
|  | *Total* | | *444* | | *226* | *218* | *52* |  |  |

^1^ the number of collections with three stages are include in tick presence

Table 2. Years lag between prediction and detection of tick or three stages by active surveillance in municipalities. Mean, range and standard deviation per municipality according to lower, mean and upper predicted year.

|  | **Year Lag between prediction and observation (mean, range, sd)*** | | |
| --- | --- | --- | --- |
|  | **Lower year** | **Mean year** | **Upper year** |
| **Three stages** | 5.69 (-1;10)  Sd=2.71 | 3.32 (-5;8)  Sd=2.91 | -0.19 (-9;5)  Sd=3.31 |
| **Tick presence** | 5 (-7;12)  Sd=3.40 | 2.4 (-12;9)  Sd=3.73 | -1.26 (-19;5)  Sd=4.16 |

*Year lag = observation year – predicted year; range= (minimum;maximum); sd = standard deviation

**Concordance over the study period.**

- **Detection of three stages.** The probability to have a known established tick population in a municipality in 2018 - by the criterion of detecting the three stages by active surveillance between 2010 and 2018 - was significantly higher if the model predicted tick establishment before 2018 (Table 3). The three stages were detected in 20% (35/171) of municipalities with a mean predicted year before 2018, but 0% (0/60) of municipalities with a mean predicted year after 2018 (16% vs 0% for lower year and 34% vs 4% for upper year; p<0.001). The agreement between the detection of the three stages and prediction was significant, but better with the upper predicted year (kappa = 0.20 [95%CI: 0.11-0.29]).
- **Detection of at least one tick.** We observed the same trends as with the three stages. Ticks were detected in 68% (115/171) of municipalities with mean predicted year before 2018 – which means predicted established tick population during the period - and in 12% (7/60) of municipalities with mean predicted year after 2018 (74% vs 29% for lower predicted year and 58% vs 10% for upper predicted year; p<0.001). The agreement between tick observation and prediction is significant, but better with the mean or upper predicted year (kappa = 0.47 [95%CI: 0.36-0.59]) (table 3).

**Table 3. Concordance between field data and predictions by 2018. Number and percentage of municipalities (CSD) based on surveillance results (no detection, tick presence, three stages) in 2010-2018 and prediction for 2018 (before or after 2018, for lower, mean and upper predicted year)**.

|  |  | | **Number (%) of CSD with tick collection in 2010-2018 (n=231)** | | | | **Kappa [IC 95%]**  **A: Tick presence**  **B: Three stages** | **McNemar test**  **p-value** |
| --- | --- | --- | --- | --- | --- | --- | --- | --- |
|  | | **Established tick pop** (Predicted year) | **Total** | **No detection** | **Tick presence** | **Three stages^1^** |  |  |
| **Lower predicted year** | **Expected** (Before 2018) | | 209 | 89 (43%) | 120 (57%) | *35 (16%)* | A: 0.17 [0.09-0.25] | <0.001 |
|  | **Not expected** (After 2018) | | 22 | 20 (91%) | 2 (9%) | *0 (0%)* | B: 0.03 [0.02-0.06] | <0.001 |
| **Mean predicted year** | **Expected** (Before 2018) | | 171 | 56 (32%) | 115 (68%) | *35 (20%)* | A: 0.43 [0.33-0.54] | <0.001 |
|  | **Not expected** (After 2018) | | 60 | 53 (88%) | 7 (12%) | *0 (0%)* | B: 0.11 [0.07-0.16] | <0.001 |
| **Upper predicted year** | **Expected** (Before 2018) | | 118 | 28 (24%) | 90 (76%) | *30 (25%)* | A: 0.47 [0.36-0.59] | <0.001 |
|  | **Not expected** (After 2018) | | 113 | 81 (72%) | 32 (28%) | *5 (4%)* | B: 0.20 [0.11-0.29] | <0.001 |
| ***Total*** |  | | *231* | *109 (47%)* | *122 (53%)* | *35 (14%)* |  |  |

1 *the number of collections with three stages are include in tick presence*

**Analysis of projection in Quebec**

- **Speed of tick spread.** For the period 2020-2100, the overall speed of tick progression calculated by the time-to-establishment model (using the mean predicted year) is on average 18 km/year for the 1,180 municipalities in Québec (figure 2). The speed was similar with upper and lower predicted year (15 and 23 km/year), but there was a six-year lag between the upper/lower predicted year and the mean predicted year. For the 2020-2030 projections, tick progression was 22 km/year using the mean predicted year (14 and 21 km/year for the lower and upper predicted year). We noted a relatively homogeneous increase over time, except for two strong accelerations in the 2046-2047 and 2067-2068 projections, which correspond to the prediction of tick population establishment in large municipalities. Removing these areas did not significantly change speed estimation.
- **Exposed human population.** According to the model predictions and based on the mean predicted year, 58% of the Québec population would live in an established tick area by 2012, 85% by 2020 and 90% by 2027 (respectively 2018, 2026 and 2032 with upper predicted year) (figure 2). There is, on average, a 3-year time lag between mean and lower/upper predictions.

Figure 2. Predicted progression of tick establishment area and human population exposed to tick in Québec from 2008 to 2100 according to the model. In solid lines, the percentage of the Quebec human population living in an area with established tick populations, according to the lower, mean, and upper predicted year (colored in light, medium, and dark grey, respectively). In dashed lines, the area of Quebec province with established tick populations (expressed as a percentage) according to the lower, mean and upper predicted year (colored in light, medium and dark grey, respectively). The speed of tick progression is represented by the slope of the curves.

- **Regional specificity.** Predictions up to 2100 show a northern expansion of tick populations from southern Québec and along the St. Lawrence River (figure 3). However, in some municipalities, tick establishment varies from surrounding municipalities, with cluster of earlier or later tick establishment. Most regions currently affected by Lyme disease have established tick populations predicted by the model. In the other regions, tick establishment should be expected in the coming years.

Figure 3. Prediction of tick population establishment in Québec from 2008 to 2100 by 5-year intervals. Predicted year of tick population establishment per municipality based on the upper predicted year of the model of Leighton et al. (2012).

**DISCUSSION**

In our study, we showed that the “time-to establishment” model of Leighton et al. (19) gave a good trend of the presence of at least one *I. scapularis* tick and the establishment of a tick population reported by field data from active surveillance from Québec, for the period 2010-2018. Concordance between observation and prediction was relatively low but statistically significant, with no major geographical incoherence. The mean predicted year seems to be a good predictor of tick presence in active surveillance; the upper predicted year, a better predictor of the three stages detection in active surveillance. The predictions correctly reflect the known epidemiological situation in 2018, and the model seems sufficiently reliable to estimate the progression of tick populations to 18 km/year for Quebec province. The model suggests a northern expansion of ticks in the coming decades, and that 90% of Quebec’s population will live in a tick-endemic area and be exposed to ticks by 2032.

**Lyme disease risk is associated with the presence of an established tick population** (28–30), **which is difficult to define.** One goal for the public health authorities is to predict where and when tick populations will establish to inform and protect human populations. Established tick populations reproduce locally, but different criteria are used to define this. The first Canadian definition of established tick populations was when all three tick life stages were detected during at least two consecutive years (12). However, this definition required significant human and financial resources to repeatedly collect ticks in the environment during two seasons. In Québec, the province used the detection of all three tick stages within the same year (6) to define an established tick population. However, Quebec also used the criterion of “the presence of at least one tick” to determine Lyme disease risk level per municipality (5). The model developed by Leighton et al. (19) was designed to predict the year of tick population establishment, based on the detection of at least two adult ticks or one immature tick, by active surveillance for two consecutive years. These definitions of tick population establishment are not currently used by Quebec’s public health authorities to assess the risk of Lyme disease. We decided to compare these two definitions with model predictions in order to understand how to use this model in public health in Quebec.

**Concordance between field data and model predictions is low but statistically significant, probably as a consequence of limits inherent to active surveillance and modelling tools.** On the one hand, the sensitivity of the dragging method to detect ticks is low because the efficiency of dragging depends on meteorological conditions during sampling (13,26) and because ticks have a heterogeneous distribution at a fine geographical scale (30–34). Detection of larvae, nymph and adults of *I. scapularis* during the same visit is highly unlikely since they are present during different seasons (11). Not observing a tick during a single visit to a municipality may also lead to the incorrect conclusion of absence. On the other hand, detection of any tick stage, especially if only one tick is found, does not always indicate an established tick population. Instead, such observations could be adventitious ticks brought by vertebrate hosts (migratory birds, rodents, deer) into areas without established tick populations (32,35,36). In our active surveillance data, for 30% of municipalities with a first tick detection, ticks were not always detected during sampling in subsequent years. These limits likely explain the low concordance we observed, especially collection by collection, and why our results are more consistent when cumulating surveillance data over the study period. These limits also explain the better results with the criterion of “presence of at least one tick” rather than the criterion of “presence of three stages”, which is harder to attain with active surveillance methods in Quebec. Moreover, active surveillance is not carried out every year in all municipalities in Québec; this incomplete sampling underestimates tick establishment and overestimates the time lag between the predicted year of establishment and detection of ticks by field sampling (three years on average in our study). It would also be interesting to conduct further studies with tick sampling outside the current active tick surveillance area to validate projections in the next decades.

**Some divergence between predictions and field data may be explained by factors that may influence tick progression**, such as forest type, micro-climate, and distribution and behavior of vertebrate hosts (including humans). These factors were not included in the model of Leighton et al. (19). This could explain some divergence between predictions and observations, as well as the real progression of ticks over the next decade. Temperature is supposed to be the most important factor to determine suitable areas for tick establishment (16,37), but it could be different in northern Québec where the habitat is less deciduous forest and where vertebrate host communities are different from southern Québec. Leighton et al. (19) suggested that rainfall and elevation may also influence tick population establishment within some regions. Moreover, previous studies conducted at finer spatial scales highlighted the heterogeneous distribution of ticks (30–34), probably as a result of differences in local conditions and host communities that support local and long-distance dispersion of ticks. Therefore, geographic agreement between active surveillance and the predictive map enhances the global suitability of prediction. Thus, municipalities with detection of the three tick stages, but outside of the predicted area, remain very close to the model boundary. Moreover, we noticed local particularities in the predictive map (e.g., local area with tick establishment earlier or later than surrounding area) which were also observed in the acarological surveillance data (results not presented here). Predictions by the model give a global picture of the tick establishment area, even if some smaller scale variations are expected within this area.

**Because field observations were consistent with model predictions, we explored the speed at which tick population establishment progressed across Quebec**. The overall speed of tick progression is estimated to be 18 km/year [95% CI; 15 – 23 km/year], which is lower than the value of 46 km/year for eastern Canada suggested by Leighton et al. (19). The original model was for all eastern Canadian provinces (i.e., Québec, Ontario, etc.), but provincial data were not detailed in the publication. However, the study carried out in southern Ontario agreed with the speed of tick progression of 46 km/year (20), by comparing the predicted year of establishment of a tick population within a radius of 46 km from a site with an established tick population. Using a model based on degree days > 0°C (16,17) and tick abundance, Simon et al. (18) estimated a northward expansion of *I. scapularis* of 300 km from 2011 to 2050 in southern Quebec, representing a speed of tick progression of 7 km/year, which is more consistent with the estimate from the present study. The model by Simon et al. (18) was based on environmental variables and was not biased by the administrative divisions, such as municipalities, which can artificially increase estimates of the speed of tick progression, as we have seen in our study with larger municipalities in northern Québec. Moreover, the real speed of establishment also depends on suitable habitat and the presence of host communities. This could change with climate change and its effects on the different components of tick ecology.

**Application in public health.** Despite the aforementioned limitations, our model reliably estimated the trends of tick progression, and by extension the risk of human Lyme disease, for the next decades. The presence of at least one tick detected by active surveillance is a criterion of an “at-risk” municipality in Québec (5) and may reflect the presence of adventitious ticks brought by migratory birds or potentially established tick populations. The detection of the three tick stages by active surveillance and during the same season, with detection of *B. burgdorferi* in at least one nymph, reflects the presence of an established tick population and determines an “endemic” municipality in Québec (5). Consequently, the mean predicted year could be used as an indicator of the “at-risk area” while the upper predicted year could be used as an as indicator of the “endemic area”, currently and for the coming decades. This approach helps to estimate the present and future risk levels of all municipalities in Québec, and could guide future active surveillance activities. Since the distribution of *B. burgdorferi* is expected to follow a similar trajectory to that of *I. scapularis* (22), the projection maps could anticipate future changes in the epidemiology of Lyme disease in Québec. The tick distribution gives an estimate of the exposed human population, with potentially 90% of Québec’s population living in an endemic area by 2032, because most of human population lives in southern Québec and along the St. Lawrence River. This estimate could be refined using the demographic projections for Quebec. Even though other factors influence the number of human cases of Lyme disease (e.g., human behavior, prevention, diagnosis) (4), these predictions could help public health authorities develop preventive measures, particularly in regions not yet affected by Lyme disease.

**CONCLUSION**

Our study shows that we can use a predictive model to estimate the risk of Lyme disease at the municipality level in Québec for the coming years. Assessing the concordance between a predictive model and field data is an important step in using prediction tools for public health authorities. This model should be updated as Québec’s field data increases in the coming years and could be validated in other Canadian provinces.

**ACKOWLEDGEMENTS**

We thank all the people involved in tick collection in the field as part of the active tick surveillance. This research was supported by the Public Health Agency of Canada (PHAC), the Institut national de santé publique du Quebec (INSPQ), and the Epidemiology of Zoonoses and Public Health Research Unit (GREZOSP), University of Montreal. Thanks to Julie Ducrocq as an external reviewer.

**REFERENCES**

1.	Burgdorfer W, Barbour A, Hayes S, Benach J, Grunwaldt E, Davis J. Lyme disease-a tick-borne spirochetosis? Science. 1982;216(4552):1317‑9.

2.	Public Health Agency of Canada. Surveillance de la maladie de Lyme [Internet]. 2020. Disponible sur: https://www.canada.ca/fr/sante-publique/services/maladies/maladie-lyme/surveillance-maladie-lyme.html

3.	Bouchard C, Leonard E, Koffi JK, Pelcat Y, Peregrine A, Chilton N, et al. The increasing risk of Lyme disease in Canada. Can Vet J. 2015;56(756):693‑9.

4.	Bouchard C, Dibernardo A, Koffi J, Wood H, Leighton P, Lindsay L. Increased risk of tick-borne diseases with climate and environmental changes. Can Commun Dis Rep. 2019;45(4):83‑9.

5.	Institut national de santé publique du Québec. Proposition d’un programme de surveillance intégré pour la maladie de Lyme et les autres maladies transmises par la tique - Mise à jour 2015. Montréal, QC, Canada; 2015 p. 45.

6.	Institut national de santé publique du Québec. Proposition d’un programme de surveillance intégré pour la maladie de Lyme et les autres maladies transmises par la tique. Montréal, QC, Canada; 2014 p. 95.

7.	Ministère de la santé et des services sociaux. Maladie de Lyme. Tableau des cas humains - Archives 2014 à 2019 [Internet]. 2020. Disponible sur: https://www.msss.gouv.qc.ca/professionnels/zoonoses/maladie-lyme/tableau-des-cas-humains-lyme-archives/

8.	Institut national de santé publique du Québec. Rapport de surveillance de la maladie de Lyme : année 2017. Montréal, QC, Canada; 2018.

9.	Institut national de santé publique du Québec. Résultats de surveillance de la maladie de Lyme : année 2018. Montréal, QC, Canada; 2019.

10.	Institut national de santé publique du Québec. Résultats annuels de surveillance de la maladie de Lyme. Année 2019. Montréal, QC, Canada; 2020.

11.	Piesman J, Gern L. Lyme borreliosis in Europe and North America. Parasitology. 2004;129(7):S191‑220.

12.	Health Canada. Consensus conference on Lyme disease. Can Med Assoc J. 1991;144:1627‑32.

13.	Ogden N, Koffi J, Lindsay L. Assessment of a screening test to identify Lyme disease risk. 2014 p. 1‑5.

14.	Institut national de santé publique du Québec. Rapport de surveillance de la maladie de Lyme : année 2015. Montréal, QC, Canada; 2016 p. 10.

15.	Institut national de santé publique du Québec. Rapport de surveillance de la maladie de Lyme et des autres maladies transmises par la tique Ixodes scapularis au Québec entre 2004 et 2013 Sommaire. Montréal, QC, Canada; 2016 p. 8.

16.	Ogden NH, Bigras-Poulin M, O’Callaghan CJ, Barker IK, Lindsay LR, Maarouf A, et al. A dynamic population model to investigate effects of climate on geographic range and seasonality of the tick Ixodes scapularis. Int J Parasitol. 2005;35(4):375‑89.

17.	Ogden NH, St-Onge L, Barker IK, Brazeau S, Bigras-Poulin M, Charron DF, et al. Risk maps for range expansion of the Lyme disease vector, Ixodes scapularis, in Canada now and with climate change. Int J Health Geogr. 2008;7(1):24‑24.

18.	Simon J a, Marrotte RR, Desrosiers N, Fiset J, Gaitan J, Gonzalez A, et al. Climate change and habitat fragmentation drive the occurrence of Borrelia burgdorferi, the agent of Lyme disease, at the northeastern limit of its distribution. Evol Appl. 2014;7(7):750‑64.

19.	Leighton P, Koffi JK, Pelcat Y, Lindsay LR, Ogden NH. Predicting the speed of tick invasion: an empirical model of range expansion for the Lyme disease vector Ixodes scapularis in Canada. J Appl Ecol. 2012;49(2):457‑64.

20.	Clow K, Leighton PA, Ogden NH, Lindsay LR, Michel P, Pearl DL, et al. Northward range expansion of Ixodes scapularis evident over a short timescale in Ontario, Canada. Wooten RM, éditeur. PLOS ONE. 2017;12(12):e0189393.

21.	Statistics Canada. Census Profile, 2016 Census [Internet]. 2019. Disponible sur: https://www12.statcan.gc.ca/census-recensement/2016/dp-pd/prof/index.cfm

22.	Ogden NH, Bouchard C, Kurtenbach K, Margos G, Robbin Lindsay L, Trudel L, et al. Active and passive surveillance and phylogenetic analysis of Borrelia burgdorferi elucidate the process of lyme disease risk emergence in Canada. Environ Health Perspect. 2010;118(7):909‑14.

23.	Keirans JE, Clifford CM. The genus Ixodes in the United States: a scanning electron microscope study and key to the adults. J Med Entomol Suppl. 20 juill 1978;2:1‑149.

24.	Keirans JE, Hutcheson HJ, Durden LA, Klompen JS. Ixodes (Ixodes) scapularis (Acari:Ixodidae): redescription of all active stages, distribution, hosts, geographical variation, and medical and veterinary importance. J Med Entomol. mai 1996;33(3):297‑318.

25.	Ogden NH, Lindsay LR, Beauchamp G, Charron D, Maarouf A, O ’callaghan CJ, et al. Investigation of relationships between temperature and developmental rates of tick Ixodes scapularis (Acari: Ixodidae) in the laboratory and field. J Med Entomol. 2004;41(4):622‑33.

26.	Daniels T, Falco RC, Fish D. Estimating population size and drag sampling efficiency for the blacklegged tick (Acari: Ixodidae). J Med Entomol. 2000;37(3):357‑63.

27.	R Development Core Team. R: A Language and Environment for Statistical Computing. 2020.

28.	Diuk-Wasser MA, Vourc’h G, Cislo P, Hoen AG, Melton F, Hamer S a., et al. Field and climate-based model for predicting the density of host-seeking nymphal Ixodes scapularis, an important vector of tick-borne disease agents in the eastern United States. Glob Ecol Biogeogr. 2010;19(4):504‑14.

29.	Daniels TJ, Boccia TM, Varde S, Marcus J, Le J, Bucher DJ, et al. Geographic risk for lyme disease and human granulocytic ehrlichiosis in southern New York state. Appl Env Microbiol. 1998;64(12):4663‑9.

30.	Pardanani N, Mather TN. Lack of spatial autocorrelation in fine-scale distributions of Ixodes scapularis (Acari: Ixodidae). J Med Entomol. 2004;41(5):861‑4.

31.	Ripoche M, Lindsay LR, Ludwig A, Ogden NH, Thivierge K, Leighton PA. Multi-scale clustering of Lyme disease risk at the expanding leading edge of the range of Ixodes scapularis in Canada. Int J Environ Res Public Health. 2018;15(4).

32.	Wilson ML. Distribution and abundance of Ixodes scapularis (Acari: Ixodidae) in North America: ecological processes and spatial analysis. J Med Entomol. 1998;35(4):446‑57.

33.	Ostfeld RS, Hazler KR, Cepeda OM. Temporal and spatial dynamics of Ixodes scapularis (Acari: Ixodidae) in a rural landscape. J Med Entomol. 1996;33(1):90‑5.

34.	Ostfeld R, Glass GE, Keesing F. Spatial epidemiology: an emerging (or re-emerging) discipline. Trends Ecol Evol. 2005;20(6):328‑36.

35.	Ogden NH, Lindsay LR, Hanincová K, Barker IK, Bigras-Poulin M, Charron DF, et al. Role of migratory birds in introduction and range expansion of Ixodes scapularis ticks and of Borrelia burgdorferi and Anaplasma phagocytophilum in Canada. Appl Environ Microbiol. 2008;74(6):1780‑90.

36.	Scott JD. Studies abound on how far north Ixodes scapularis ticks are transported by birds. Ticks Tick-Borne Dis. 2016;7(2):327‑8.

37.	Lindsay LR, Mathison SW, Barker IK, McEwen SA, Gillespie TJ, Surgeoner GA. Microclimate and habitat in relation to Ixodes scapularis (Acari: Ixodidae) populations on Long Point, Ontario, Canada. J Med Entomol. 1999;36(3):255‑62.

**Supporting information**

Access to Quebec data surveillance needs a formal request to government of Quebec. Access to model prediction needs a request to the authors of the publication of Leighton et al. (2012)
