# Supplementary material for: Current and future distribution of Ixodes scapularis ticks in Québec: Field validation of a predictive model
Source: PLoS One. 2022 Feb 3;17(2):e0263243. doi: 10.1371/journal.pone.0263243 (PMC8812838; doi:10.1371/journal.pone.0263243)
Supplement: Attachment — Submitted filename: 20211004_Response to Reviewers.doc [file pone.0263243.s003.doc]

Dear reviewers,

Thanks for your pertinent comments and suggestion, it was a real contribution for the manuscript. Thank you for your understanding about my English, which is unfortunately far from perfect, and for making the effort to overpass or correct it by reviewing the article, it is much appreciated.

You will find below and in the revised manuscript the response to your comments.

**Review Comments to the Author**

Please use the space provided to explain your answers to the questions above. You may also include additional comments for the author, including concerns about dual publication, research ethics, or publication ethics. (Please upload your review as an attachment if it exceeds 20,000 characters)

**Reviewer #1**: Title: Current and future distribution of Ixodes scapularis ticks in Québec: field validation of a predictive model previously developed in Canada

Authors: Marion Ripoche, Catherine Bouchard, Alejandra Irace-Cima, Patrick Leighton, Karine Thivierge

Summary of the study: The spread of Ixodes scapularis ticks into Canada and the resultant increase in the incidence of Lyme borreliosis in the Canadian population is a well-documented example of how climate change can increase the public health burden of vector-borne diseases. A model by Leighton and colleagues predicted the speed at which blacklegged ticks would expand their range in eastern Canada and establish new tick populations (1). The purpose of the present study is to test how this model performs in southern Quebec where the authors have spent 9 years (2010 – 2018) doing active surveillance for blacklegged ticks by using the dragging method. The authors conducted 444 tick sampling sessions in 231 census subdivisions (CSDs) between 2010 and 2018. The authors used two different criteria to determine whether a particular site had an established tick population: (1) sampling of all three tick stages (strict definition) and (2) sampling of a single blacklegged tick (less restrictive definition). The authors used the model by Leighton and colleagues to predict the year of blacklegged tick population establishment for each of the sampling sessions and CSDs (they used a mean, lower, and upper estimate). They then compared these predicted values (3 predictions) to the observed values from their active surveillance (2 criteria for an established tick population). The general result was that active surveillance was more likely to find ticks in sites that were predicted to have established tick populations according to the model compared to sites that were not predicted to have established tick populations. For the 444 sampling occasions, the model based on the mean year predicted 65.1% correctly (289/444) and 34.9% (155/444) incorrectly (Table 1). For the 231 CSDs, the model based on the upper year predicted 74.0% correctly (171/231) and 26.0% (60/231) incorrectly (Table 3). The model predicts that 90% of the human population in Quebec will be leaving in an area with an established tick population by 2027 (prediction based on the mean).

Major comments:

-The authors have used a straightforward approach to answering their question, and I don’t have too many criticisms of the study.

MR:ok

-The authors show that active surveillance is more significantly more likely to find ticks in areas that were predicted to have established tick populations compared to sites that were not. However, it would be useful if the authors came up with an overall measure of the ability of their model to predict the correct answer. For example, in Table 1 for the lower predicted year, the model predicted 55.4% of the sampling occasions correctly [(199 + 47)/444 = 246/444] and 44.6% of the sampling occasions incorrectly [(179 + 19)/444 = 198/444]. In other words, for this particular combination of prediction (lower year) and outcome (1 tick present is an established tick population), the model is not much better than flipping a coin. It would be good if this was pointed out in the manuscript. For the 231 CSDs, the model does better and for the upper year, it predicts 74.0% (171/231) of the CSDs correctly and 26.0% of the CSDs incorrectly (60/231). Again, it would be good to point out that for this combination of prediction (upper year) and outcome (1 tick present is an established tick population), the model performs better than a random coin toss.

MR:I agree, it is clearer like that, I added that in the result and discussion.

-In the Discussion, the authors point out that the original model by Leighton and colleagues (1) predicted a speed of progression of established tick populations of 46 km per year. The estimated speed in the current study is considerably slower, 18 km per year over the time period of 2020 – 2100 and 22 km per year over the time period 2020 – 2030. A study in southern Ontario confirmed the speed of progression of established tick populations of 46 km per year, whereas a study in southern Quebec found a speed of progression of 7 km per year. In the Discussion, it is not really clear why the speed of progression differs between Quebec and Ontario, and some more clarification would be helpful.

MR: We calculated the speed with the data from the model. I don’t know the predicted speed for Ontario.

-In the Discussion, the authors discuss the application of their model for public health. However, according to their model predictions, 90% of the human population of Quebec will be living in an area with an established tick population by 2027. If this prediction comes true, what is there left to say for public health authorities other than that almost the entire population in Quebec is at risk for Lyme disease and ask everyone to be careful?

MR:It is true. But it is important to explain to region with no established tick population for the moment, that it will be coming in the next decades, even for region in the north with a rude winter

-The manuscript was formatted with bullets. Is this a new formatting requirement for PLOS ONE manuscripts? If not, please don’t use bullets, as it gives reviewers the impression that the manuscript was hastily prepared.

MR:the bullet was to mark the section and sub-section. I deleted them.

-The manuscript contained many small grammatical errors. The authors should do a better job of checking the manuscript for such errors before submitting it to a journal. I have attached a Word document with ‘Track changes’ that documents a number of these grammatical errors.

MR: Thanks for having corrected grammatical errors. I really appreciate that, and I will find a way to check before submission the next time

Advice for future studies:

-The authors point out that the dragging method does not always detect the ticks at a particular site, even if the site is believed to have an established tick population. In future studies, the authors should consider using site occupancy models to address this problem. For example, you may sample the same site in 2016, 2017, and 2018, and find ticks in 2016 and 2018, but not in 2017. If we assume that the ticks were there in 2017, but were not detected, we can estimate the probability of not detecting ticks at a site with an established tick population. By separately estimating the probability of detection versus the probability of an established tick population, you will get better estimates of the latter. In Table 1, the best model (based on the mean year) predicts 65.1% of the sampling occasions correctly, whereas in Table 3, the best model (based on the upper year) predicts 74.0% of the CSDs correctly. This comparison suggests that failing to detect ticks in a site where they are established is less of a problem for the CSDs than the sampling occasions. This observation makes sense, because there are multiple sampling occasions for each CSD, which reduces the error due to the failure of detecting ticks at a site where they are established. If you conducted a very large number of sampling occasions at each site (e.g., hundreds), you would eventually have perfect knowledge of which sites had established tick populations and which did not.

MR: I agree with you, but active surveillance in Quebec is currently limited to one visit per municipality and per year, and aa we are note able to visit all municipality each year, there is a turn over. But we have some sentinels sites, sampled each year, that could be used to estimate the probability of detection or not detection in a site with established tick population.

-The authors do not give much insight as to why their model is not working better. They point out that a number of factors are not included in the original model by Leighton and colleagues (1). For example, the model is heavily reliant on temperature, but does not include elevation and rainfall. This study would have been more interesting if the authors had dropped components from the original model to quantify the contribution of those components to the accuracy of the predictions. Thus, an unsatisfying feature of this study is that the authors and the readers don’t really learn anything new about how the model by Leighton et al. could be improved. If the authors plan on future studies investigating the predictive ability of the model by Leighton et al., they should consider comparing different sub-models that drop different components of the original model, so that we learn which components of the models are actually important for making the correct predictions.

MR:Yes, in further study we could try to improve the model with more recent environemental and surveillance data than in the original model.

References

1. Leighton PA, Koffi JK, Pelcat Y, Lindsay LR, Ogden NH. 2012. Predicting the speed of tick invasion: an empirical model of range expansion for the Lyme disease vector Ixodes scapularis in Canada. J Appl Ecol 49:457-464.

**Reviewer #2**: GENERAL REMARKS

The study validates a predictive model on tick distribution in Canada, by comparing collected field data with the model predictions. These types of model evaluations are useful to assess and improve on the predictive model. However, although I would consider this study to be worthwhile, there are many linguistic errors to be addressed before the publication of this manuscript. Please find more specific remarks below.

ABSTRACT

If the use of title words such as Background, Objective etc. was a conscious decision, these should be clearer. Perhaps adding “:” or a line break can make this distinction between different subheadings more readable.

Line 26: “The incidence of Lyme disease”, or “The recorded number of Lyme disease cases” is increasing in Québec…
Line 27: remove “,” after “2012”
Line 28: put “until 2100” after “establishment”
Line 44: I would add “Ixodes scapularis” to the list of key words

MR: done – changed in the text

INTRODUCTION

Line 49: Locally acquired cases (per year? In total?) please specify. You could say “increased from 144 annual cases in 2009, to 2636 in 2019”. Put “in Canada” at the beginning of the sentence.
Line 51: “is progressing”
Line 58: check commas and punctuation in general
Line 77: “Active acarological surveillance”
Line 95: all three stages

MR: done – changed in the text


METHODS

Study area: As this is a study assessing the viability of a model based on climatic data: Some general climatic information (mean yearly temperatures, precipitation), would be welcome. This is also true for the elevational range (as this is factored into the model), and overall vegetation composition (what type of forests).

MR:

Some general information on I. scapularis would be welcome, including the vectorial capacity of this species, as well as its ecology (questing behavior and host-parasite relations).

MR: changed in introduction l. 52-58

Line 104: “province”
Line 117: tick distribution “data”, comes from …
Line 123: which configuration? Lines, quadrats, one single 2000m line? Specify
Line 130: spacing after citation
Line 131: year of establishment
Line 132: years

MR: done – changed in the text

Line 139: “tick populations were”, or “a tick population was” supposed to be established
Line 138: remove first “of the model”, remove first comma
Line 146: “A tick population” or “tick populations”, there where and when…
Line 147: Predicted for it
Line 151: “if a tick population is predicted to be established in the area, then ticks are also expected to be found in the field data”
Line 169: for clarity, I suggest inserting a full equation in word in the form of:
Speed[km/year]=√(area*year*n)- √(area*year*n-1)

MR: done – changed in the text

Line 172: “exposed to ticks”, remove first comma,
Line 173: “predicted an established tick population”
Line 175: “across the population of Quebec”
Line 176: “…of established tick populations”, “was produced/generated”

MR: done – changed in the text

RESULTS

Line 184: add percentage
Line 199: “twice on average”
Line 209: “Concordance per collection”

MR: done – changed in the text

Table 1: Is there a reason why the three stages column data is in italic? If so, specify.

MR: yes, because the number of collections with three stages are included in tick presence (cf. footnote of table), it’s a sub-part of collection with tick presence

Table 2: If you use “Sd” in the table, the *text should be “Sd” as well, not “sd”.
Table 1 and 3: footnote: “are included”
Line 240: remove second “detection”
Line 242: “the three stages”
Line 245-246: If you include the statistical result, please also include the p-value

MR: done – changed in the text

DISCUSSION

I am missing some recommendations on if/how the findings of this study (field validation) could be used to enhance the model developed by Leighton (2012).

MR: added in the text Line 430

Line 298: “time-to-establishment”
Line 310: where and when “A tick population” or “tick populations” will establish
Line 315: Collect ticks
Line 320: Years
Line 321: remove comma. Risk of what? Specify Lyme disease risk
Line 322: “with the model predictions”
Line 338-339: Because of “limitations” in “logistical” capacity
Line 349: “influential factor”

Line 350: northern, sometimes “Quebec” is written “Québec”. Please be consistent.

MR: done – changed in the text

Line 351: host communities are different how?

MR: vertebrate host communities are different from southern Québec

Line 352: “A previous study”, or “previous studies”
Line 363: remove comma

MR: done – changed in the text
