# Supplementary material for: Current and future distribution of Ixodes scapularis ticks in Québec: Field validation of a predictive model
Source: PLoS One. 2022 Feb 3;17(2):e0263243. doi: 10.1371/journal.pone.0263243 (PMC8812838; doi:10.1371/journal.pone.0263243)
Supplement: Attachment — Submitted filename: 20211215_PlosOne_Response to Reviewers.docx [file pone.0263243.s004.docx]

6. Review Comments to the Author

Please use the space provided to explain your answers to the questions above. You may also include additional comments for the author, including concerns about dual publication, research ethics, or publication ethics. (Please upload your review as an attachment if it exceeds 20,000 characters)

**Reviewer #2:**

GENERAL REMARKS
The proposed revised manuscript is a substantial improvement on the initial submission. Particularly, the discussion now thoroughly outlines the limitations of the findings and the methods, and how these may be improved in the future. Overall, I would consider this manuscript fit for publication, if a few minor changes are implemented.
Line 1: “TITLE”

MR: done
INTRODUCTION
Line 51: “climatic” conditions
Line 52: …deciduous and mixed forests are “considered” suitable “habitats”…
Line 54: remove comma
Line 61: …drag sampling “method”.
Line 76: “northwards”

MR: done
Line 97 and lower: If chosen to make et al. italic, please do so throughout the manuscript.

MR: done
METHODS
Line 128-129: During the initial review, a request was made to specify the configuration of the 2000m2 area that was sampled using the flag dragging method. This was not done. It would be suitable to specify whether the sampling was done in simple line of 2000m, or if quadrats were used and if so, in which sampling structure.

MR: done. I added explanation in text, line 130-131
Line 155-156: This question is repeating the same declarative statement of the sentence before. I suggest removing this question.

MR: I prefer to keep the question to clarify for the lector, explaining the analysis in another way
Line 184: Remove empty line to keep the spacing consistent with the other chapters

MR: I standardized between sections
RESULTS:
Figure 2: Having black x and y labels would greatly enhance readability.

MR: done
DISCUSSION
Of all the chapters, the discussion has improved the most compared to the initial submission.

MR: Thanks
Line 368: “limitations”

MR: done

**Reviewer #3:**

This study uses field data to validate a time-to-establishment model developed in 2012 by one of the authors to predict the Ixodes scapularis population establishment for each municipality in eastern Canada. This field data consisted of active tick surveillance data collected betweem 2010-2018, where two criteria were used to define tick establishment: i) either the detection of at least one tick or ii) the detection of the three questing stages of the tick.

Overall I really like the premise of this paper, dragging/flagging data are valuable but rarely get published, and I like that the authors have used a more creative/informative way of doing this by using it to validate a model (which in itself is also worthwhile).

MR: Thanks

However there are some potentially significant issues with the use and interpretation of statistics, and some of the methods need more detail, for example how the human population increase was calculated and exactly when flagging/dragging (it is unclear which technique was used) was performed.

MR: I tried to clarify that in the text

Introduction
The introduction does a good job of introducing the topic, highlights the importance of the paper and clearly sets out the aims of the study.
MR: Thanks

Line 48-54: All nice points! I would also argue that improved awareness of Lyme disease in Canada, and therefore improved diagnostics and reporting are also likely to have caused the number of human cases to increase.

MR: I agree with you

Line 62: “In Québec, the number of 62 locally acquired human cases has increased from 2 cases in 2008 to 381 by 2019 (7)”
Human cases of what? Tick bite or Lyme disease? Clarify, and if Lyme disease I suggest moving this to line 58 after “a notifiable human disease in Québec since 2003.” for better information flow.

MR: done.

Lines 67-68: Which months are considered spring, summer and autumn in the study area (this is important when considering the months you sampled ticks).
MR: done.

Materials and Methods
Line 107: “The province of” should not be in bold
MR: done.
Line 122-132: There is some inconsistency in the description of tick surveillance, was it dragging with a drag cloth or flagging with a flag cloth (they can target different life stages of the tick).

MR: I added details about dragging methods

How frequently was flagging/dragging performed at each site?

MR: I added details and there is also information in results, line 209-210

Line 126 you mention tick sampling in June-September, which life stages would you realistically be likely to encounter in each of these months at the study location? This information should be included as finding each of the life stages is an important underpinning of your analyses?

MR: I added details

It is very hard for me to understand how the methods for sampling event concordance (line 146-152) and concordance over the study period (line 153-163) differ. After re-reading a few times I think I understood that concordance over the study period aggregated all tick data from 2010-2018 whereas sampling event concordance looked year by year? I think it would help the reader if this was made more explicit.

MR: yes, that is the difference. I added some details in the text.

Line 165: Please clarify your use, justification, and interpretation of McNemar’s. this is not truly paired data and McNemar does not assess for concordance.

MR: done. Line 172: “A significant Chi-square test, with p<0.05, suggests that the distribution of sites with presence of ticks is not at random between areas with expected or unexpected established tick population according to the model. We used the matched McNemar test for the analysis of all the sampling because of some repeated samples at the same site”
Line 177-179: “We cumulated the annual exposed human population from 2006 to 2100, and calculated the annual proportion of the Québec population exposed, based on the 2016 census data”. Cumulated and calculated how? Please add more detail.

MR: done


Results
Line 202-204: This should be in the methods. Do I therefore understand correctly that sites were not sampled every year? In which case this also should be made more explicit in sampling event concordance (line 146-152) and concordance over the study period (line 153-163). It would be nice to have a table of when sites were sampled, with the raw tick data and what the model predicted (lower, mean and upper year).

MR: I added some details in methods. I let description of data in result. Data are available is a request to specific person.

Line 211-214: This really depends when sampling was performed (and another reason for wanting to see a table of exactly when sites were sampled and the raw tick data), as you mention in lines 67-68 the different life stages are active at very different times of year. So, if for example you happened to sample a bunch of municipalities in early spring when larvae and adults are unlikely to be active you are going to have a very different chance of finding all three life stages than if you sample in the summer, possibly biasing your results.

MR: Sites are sampling generally once, between may and September. In endemic area, we are able to catch nymph, adults and larvae in spring. In discussion, this is the main limitation of active surveillance data (line 369), and that is why we assess the concordance with presence of tick and not only presence of confirmed established tick population.

Line 306-311: It would be nice to add some further details here, for example, how many regions affected by Lyme disease? Is the coming years 1, 2, 5 or 10 years? By how much to neighbouring municipalities differ? Is there any pattern to this?

MR: I added some details in the text (Line 310). We didn’t analyse the pattern of expansion, because it was outside the scope of our study.

Figure 2: I propose changing the figure legend title to “proportion of the human population living in municipalities with established tick populations in Québec from 2008 to 2100” or similar, as any of the population could be “exposed” just by going one day for hike in a location with ticks.

MR: done


Discussion
The general content of the discussion looks good and relevant, but I am hesitant at this stage to review it more thoroughly as I have concerns with the use and interpretation of statistics, and will be happy to read this in more detail once the authors have had a chance to respond to these concerns.

Line 323-325: “Concordance between observations and predictions was relatively low but statistically significant, with no major geographical inconsistencies” – I think you have interpreted the statistics incorrectly. The matched McNemar test does not test for concordance and I don’t see how these data are truly paired? Furthermore: “If the statistical significance level (i.e., p-value) is less than 0.05 (i.e., p < 0.05), you have a statistically significant result and the proportion of X before and after Y is statistically significantly different”, suggesting the opposite to concordance.

MR: see explanation in methods. We assessed concordance of presence of tick population between prediction and observation. We tested concordance in predicted area vs unpredicted area, for random distribution (chi-square test) ans level of concordance (kappa).

7. PLOS authors have the option to publish the peer review history of their article ([what does this mean?](https://journals.plos.org/plosone/s/editorial-and-peer-review-process#loc-peer-review-history)). If published, this will include your full peer review and any attached files.


If you choose “no”, your identity will remain anonymous but your review may still be made public.


**Do you want your identity to be public for this peer review?** For information about this choice, including consent withdrawal, please see our [Privacy Policy](https://www.plos.org/privacy-policy).

Reviewer #2: No

Reviewer #3: No

[NOTE: If reviewer comments were submitted as an attachment file, they will be attached to this email and accessible via the submission site. Please log into your account, locate the manuscript record, and check for the action link "View Attachments". If this link does not appear, there are no attachment files.]

While revising your submission, please upload your figure files to the Preflight Analysis and Conversion Engine (PACE) digital diagnostic tool, <https://pacev2.apexcovantage.com/>. PACE helps ensure that figures meet PLOS requirements. To use PACE, you must first register as a user. Registration is free. Then, login and navigate to the UPLOAD tab, where you will find detailed instructions on how to use the tool. If you encounter any issues or have any questions when using PACE, please email PLOS at [figures@plos.org](mailto:figures@plos.org). Please note that Supporting Information files do not need this step.
